# Early Monitoring of Donor-Derived Cell-Free DNA in Kidney Allograft Recipients Followed-Up for Two Years: Experience of One Center

**DOI:** 10.3390/life14111491

**Published:** 2024-11-16

**Authors:** Carmen Botella, José Antonio Galián, Víctor Jiménez-Coll, Marina Fernández-González, Francisco Morales, Gloria Martínez-Gómez, Rosana González-López, María José Alegría, María Rosa Moya, Helios Martinez-Banaclocha, Alfredo Minguela, Isabel Legaz, Santiago Llorente, Manuel Muro

**Affiliations:** 1 Immunology Service, University Clinical Hospital Virgen de la Arrixaca, Biomedical Research Institute of Murcia (IMIB), 30120 Murcia, Spainmariaj.alegria@carm.es (M.J.A.); rosa.moya2@carm.es (M.R.M.); alfredo.minguela@carm.es (A.M.); 2 Nephrology Service, University Clinical Hospital Virgen de la Arrixaca, Biomedical Research Institute of Murcia (IMIB), 30120 Murcia, Spain; 3 Urology Service, University Clinical Hospital Virgen de la Arrixaca, Biomedical Research Institute of Murcia (IMIB), 30120 Murcia, Spain; gloria.martinez5@carm.es; 4 Department of Legal and Forensic Medicine, Biomedical Research Institute of Murcia (IMIB), Faculty of Medicine, Regional Campus of International Excellence “Campus Mare Nostrum”, University of Murcia, 30100 Murcia, Spain; isalegaz@um.es

**Keywords:** donor-derived circulating free DNA (dd-cfDNA), kidney transplant, graft rejection, biomarker, non-rejection (NR), antibody-mediated rejection (ABMR), borderline rejection (BR), T-cell-mediated rejection (TCMR)

## Abstract

(1) Background: donor-derived circulating free DNA (dd-cfDNA), an innovative biomarker with great potential for the early identification and prevention of graft damage. (2) Methods: Samples were collected prospectively and the study was performed retrospectively to analyze dd-cfDNA plasma levels in 30 kidney transplant patients during their post-transplant follow-up (15 days, 3, 6, and 9 months), to determine if the result could be of interest in the identification of possible adverse events, especially rejection. The aim was to verify whether the data on sensitivity, specificity, NPV, and PPV compare with reference values and creatinine values. (3) Results: We observed levels of dd cfDNA > 1% in six of nine patients with active rejection (ABMR or TCMR) and elevated values (>0.5%) in two other patients in this rejection group. Our results show low values of sensitivity = 50%, specificity = 61.11%, rejection NPV = 64.71%, and rejection PPV = 46.13% of the technique compared to reference values previously published. With respect to creatinine, only for TCRM, we observed better results for dd-cfDNA in these parameters than in creatinine. Also, our data suggest that dd-cfDNA could help to differentiate those patients with dnDSAs that are going to through rejection better than creatinine, specially at 15 d post transplant. In this study, this appears to have no positive predictive value for borderline rejection (BR) or TCMR IA. (4) Conclusions: plasma levels of dd-cfDNA could be considered an additional or alternative biomarker for graft rejection monitoring in early post-kidney transplant up to several months before its clinical presentation, especially for patients with suspected TCMR or ABMR.

## 1. Introduction

In recent years, new and interesting biomarkers that enable the monitoring of the kidney transplant recipient have emerged. That seems promising concerning the time of anticipation in detecting allograft rejection compared to renal biopsy or other classic serological/urinary markers [1].

A detailed biopsy is currently considered the “gold standard” in monitoring transplanted organs. Consequently, biopsy has evolved to become an early detection method, which should meet the fundamental criteria defined for screening procedures, which include safety and acceptability for our patients, as well as the ability to detect a clinical condition to be combated at an opportune time in order to modify its course [2]. However, due to its invasive nature, it presents essential patient safety and acceptability limitations. Comprehensive studies in the field of kidney transplantation reveal that around 1% of biopsies result in significant and different complications, with a risk of gross hematuria exceeding 3.5% [3]. In addition, thanks to the current available immunosuppressive therapies, the detection of subclinical rejection is infrequent, which has motivated many nephrology units to stop performing such routine kidney biopsies [4].

As a result, there has been growing interest in developing non-invasive strategies capable of detecting graft failure or rejection. Currently, serum creatinine and urinary indicators of renal function, such as the urinary albumin-to-creatinine ratio (UACR), fulfill this role, as they are inexpensive, relatively reliable, and are easily interpreted. However, its sensitivity and specificity in detecting damage to allografts are poor [1,5].

There are also several other non-invasive methods for diagnosing and monitoring patients suffering rejection, such as the detection of human leukocyte antigen (HLA) and non-HLA antibodies [6]; blood gene expression profiles such as Trugraf [7] or kSORT [8]; analysis of perforin, granzyme B, and inducible protein mRNA by IFN [9]; urinary chemokine levels (CXCL9 y CXCL10); proteomic and peptide signatures of rejection in urine and blood samples; enzyme-linked immunospot assay for IFN-gamma (ELISPOT) [10]; or metabolomic changes. However, these non-invasive methods generally lack sufficient scientific evidence to support their clinical use or are too expensive to implement.

In this context, donor-derived circulating free DNA (dd-cfDNA), an innovative biomarker with great potential for the early identification and prevention of graft damage, is crucial [11]. This dd-cfDNA is present in the recipient’s blood and comes from damaged cells in the transplanted kidney [12]. When graft damage occurs, dd-cfDNA from affected cells also increases in peripheral blood. In this way, identifying an early increase in dd-cfDNA levels in recipients could reduce the number of unnecessary biopsies and provide essential information to modify immunosuppressive therapy at the right time and prevent the progression of damage [13].

The considerable diagnostic and prognostic potential of this innovative biomarker has prompted its inclusion in numerous and extensive multicenter trials. Among them, the following stand out: Allosure with CareDx, Prospera with Natera and Trac with Viracor-Eurofins [14,15,16]. These trials have precisely defined the thresholds that discriminate the presence or absence of rejection (cfDNA > 1% for kidney transplant), as well as the associated values of sensitivity, specificity, negative predictive value (NPV), and positive predictive value (PPV) linked to this technique.

In the present study, we show the results of 30 kidney transplant patients in whom dd-cfDNA levels had been evaluated during their post-transplant follow-up to determine if the information provided by dd-cfDNA could be of interest in identifying possible adverse events. In addition, the aim is to verify whether the data on sensitivity, specificity, NPV, and PPV obtained in our series are consistent with the values referenced in the most consolidated literature, considering that reactive renal biopsies or clinically indicated renal biopsies are performed in our center, but prospective biopsies by protocol are not.

## 2. Materials and Methods

Our study included patients undergoing kidney transplantation between 2020 and 2023 from the University Clinical Hospital “Virgen Arrixaca” and Biomedical Research Institute of Murcia (IMIB) (Murcia, Spain) within the framework of the PI19/01194 National Research Project funded by the Carlos III Health Institute (Madrid, Spain), with Ethical Committee permission number PI1501370.

Blood samples from patients were collected prospectively at 15 days and 3, 6, and 9 months post transplant. A 10 mL tube of Streck-Cell-Free DNA BCT^®^ was removed from each patient. The plasma was then isolated using a double-centrifugation protocol at 1600× *g* for 10 min at room temperature for the first and second centrifugation. The plasma collected was frozen at −80 °C until the time of use (Table S1).

Given the high volume of patients (greater than 150) and the logistical and economic limitations, exclusion and inclusion criteria were implemented to select a sample of 30 patients representative of the population to be studied. On the one hand, an exhaustive analysis of the patients’ medical records was carried out to identify and include the maximum number of patients with rejection, suspected rejection, or other relevant adverse effects and who had completed monitoring in the first year after transplantation. However, only 12 patients with these characteristics could be included, and the study was completed with the first 18 patients enrolled in the trial who had not experienced any adverse event during their post-transplant period and had completed follow-up. Finally, dd-cfDNA levels were determined retrospectively in 30 patients undergoing kidney transplantation with a mean age of 49.84 ± 9.86 years and a gender ratio of 70% men vs. 30% women, who were classified according to their post-transplant clinical evolution, taking as a reference the Banff classification [17,18] for subsequent study. The demographic and clinical characteristics of the patients included in this study are shown in Table 1.

The extraction of dd-cfDNA from the plasma samples of the selected patients was performed using the commercial AlloSeq-cfDNA kit (CareDx, Brisbane, CA, USA) following the specifications provided by the manufacturer. The analysis, using next-generation sequencing (NGS), was carried out using the “MiSeq TM System” (Illumina, San Diego, CA, USA). NGS outputs were analyzed using the Alloseq cfDNA v.1.0 software (ASCFS 1.0).

The data and results obtained from each patient were collected in an anonymized database with coding that prevented their identification.

Data analysis was performed using the Microsoft Office Excel software and IBM SPSS Statistics v.15 software.

The analysis results of quantitative variables were expressed as the mean and standard deviation or as the median and interquartile ranges. Qualitative variables were expressed as percentages.

For the contrast of means, Student’s *t*-test of independent and paired samples was used when the distribution was normal, or the Wilcoxon and Mann–Whitney U test (post hoc) for the contrast of medians in case of non-normal distributions. The parametric ANOVA and Tukey (post hoc) test, or the non-parametric Friedman test, were used for multiple comparisons. The contrast of percentages was performed using Pearson’s chi-square test. The level of statistical significance was established for values of *p* < 0.05.

From ethical/legal aspects, this study was evaluated and approved with a favorable opinion by the Research Ethics Committee of the IMIB-Arrixaca. All participants signed an informed consent form allowing the research team to anonymously use the data and results. Finally, none of the participating members declared any conflicts of interest.

## 3. Results

Levels of dd-cfDNA (%) were determined retrospectively in 30 kidney transplant recipients from Virgen Arrixaca Clinical University Hospital. The monitoring results of this biomarker and other biochemical data on renal function and clinical evolution are shown in Table 2.

**Figure 1:**
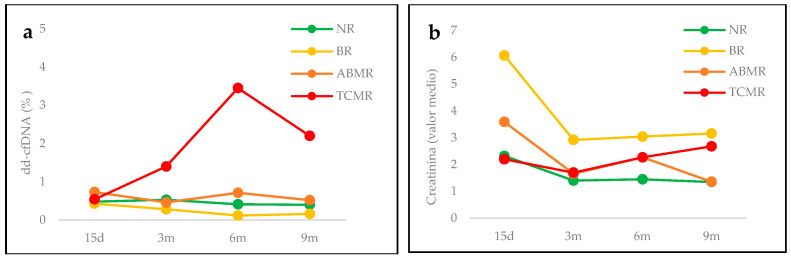
Plasma levels of (**a**) dd-cfDNA (median of %) and (**b**) creatinine (mean, mg/mL) during the post-transplantation period studied in each group. NR: non-rejection, BR: borderline rejection, ABMR: antibody-mediated rejection, TCMR: T-cell-mediated rejection.

Patients were divided into groups based on their clinical course post transplantation, and the results for each group are shown in Figure 1.

**Figure 2:**
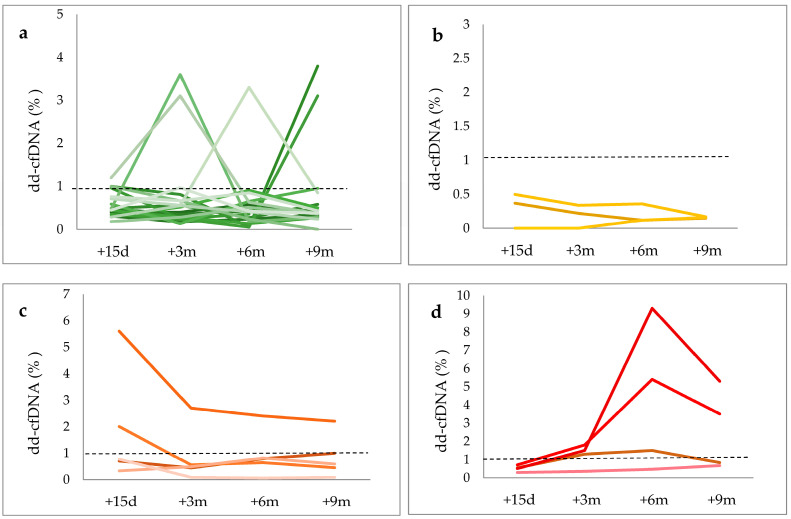
dd-cfDNA levels (%) during post-transplantation period in (**a**) patients with no signs of graft rejection (Group 1, in green colors), (**b**) patients with borderline rejection (Group 2, in yellow colors), (**c**) patients with antibody-mediated rejection (Group 3—ABMR, in orange colors), and (**d**) patients with T-cell-mediated rejection (Group 3—TCMR, in red colors).

The detailed analysis of these results for each patient included in each group and the date on rejection diagnosis are shown in Figure 2a–d.

**Group 1 (N = 18)** included patients with no signs of graft rejection without a biopsy or with a negative biopsy, who correspond to Category 1 of the Banff classification (Figure 2a), who showed means and medians of a cfDNA percentage of dd-cfDNA ≤ 1%, which represented a negative value of dd-cfDNA for kidney transplant, but only in 11 patients. The rest (n = 7) exhibited dd-cfDNA levels >1%, which other pathologies/biological processes could explain. In this sense, the analysis of seven patients with elevated dd-cfDNA values with no apparent relation to immunological causes of rejection revealed that recurrent autoimmune pathologies in the post-transplant period and other pathologies or complications like diabetes mellitus, obesity, viral infections (CMV, EBV, or HIV) and immunosuppressive drug toxicity, may influence dd-cfDNA levels. However, the existence of statistically significant differences in the variables analyzed was not demonstrated due to the small sample size.

**Group 2 (N = 3)** included patients who had borderline graft rejection (Category 3 of the Banff classification), all of them with levels of dd-cfDNA ≤ 1% (Figure 2b).

**Group 3 (N = 9)** included patients with graft rejection in both ABMR (Category 2 of the Banff classification) and TCRM (Category 4 of the Banff classification) and showed different profiles depending on the type of rejection.

In the ABMR group (N = 5), three patients showed values of dd-cfDNA > 1% in post-transplant determinations corresponding with the date-of-rejection diagnosis by kidney biopsy or clinical diagnosis with the initiation of treatment for ABMR. In one patient, values of dd-cfDNA < 1% were observed, but on the date of the rejection, the diagnosis showed an increased level of dd-cfDNA = 0.81%. The last patient in this group had an early ABMR, and the treatment was started before the first dd-cfDNA determination, so this case was considered a false negative (Figure 2c).

Regarding the TCMR group (N = 4), all patients except one showed elevated values (dd-cfDNA > −0.5%) at 15 days post transplant and positive values (dd-cfDNA > 1%) for determinations made at 3, 6, and 9 months during the post-transplant period (Figure 2d). Only one patient diagnosed with TCMR—IA by biopsy six months post transplant showed negative values of dd-cfDNA.

Moreover, most patients with TCMR showed dd-cfDNA levels >1%, anticipating the graft rejection diagnosis at least one month earlier with a positive value or an elevated value (>0.5%), as shown in Table 3. However, only in two of five patients diagnosed with ABMR were levels of dd-cf-DNA > 1% observed, coinciding with the time of rejection diagnosis, and two other patients showed values higher than 0.5 at or before the rejection date.

**Figure 3:**
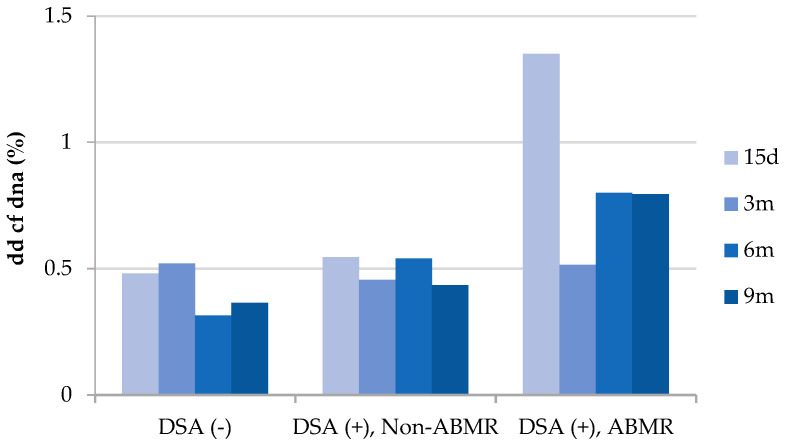
dd-cfDNA levels (%) during post-transplantation in patients considering the presence of anti-HLA DSAs and the development of ABMR.

These results of dd-cfDNA were also compared with the presence of donor-specific antibodies (DSAs) in transplant patients against HLA molecules from kidney grafts (Figure 3), and similar profiles were observed with dd cfDNA levels that barely reached 0.5% in the groups of patients who did not present DSAs and in those who had DSAs but without ABMR. However, patients with ABMR (DSAs+) showed elevated dd-cfDNA levels > 0.5% in all post-transplant determinations.

**Figure 4:**
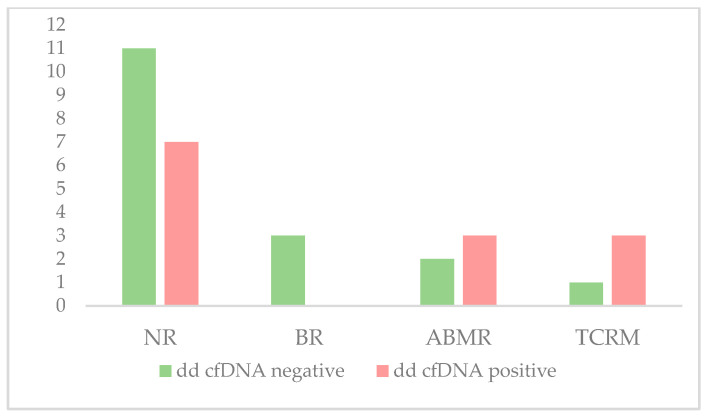
Frequencies of negative and positive patients for dd-cfDNA levels in each group. NR: non-rejection, BR: borderline rejection, ABMR: antibody-mediated rejection, TCMR: T-cell-mediated rejection.

Finally, the diagnostic validity test results are shown in Table 4 and Figure 4. Regarding overall rejection, a sensitivity of 50% was observed in our study, following the data reported in the studies carried out to date [11,12,13,14,15,16,17,18,19,20,21]. However, a specificity value of 61% was lower than those reported in the literature reviews. Accordingly, PPV and NPV were also low. However, when these parameters were analyzed for each rejection type, an NPV improvement was observed, especially in TCMR.

**Figure 5:**
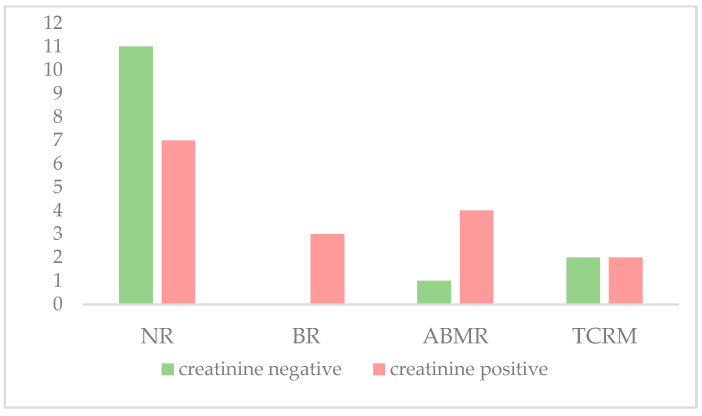
Frequencies of negative and positive patients for creatinine levels in each group. NR: non-rejection, BR: borderline rejection, ABMR: antibody-mediated rejection, TCMR: T-cell-mediated rejection.

These same analyses were performed for serum creatinine, shown in Table 5 and Figure 5.

## 4. Discussion

In the present study, we conclude that plasma levels of dd-cfDNA could be considered an additional biomarker of graft rejection for kidney transplant patients, since early-term post-transplant signs are evident up to several months before its clinical presentation, especially for TCMR patients. We have observed levels of dd-cfDNA > 1% in six of nine patients with active rejection (ABMR or TCMR) and elevated values (>0.5%) in the other two patients in this group.

Considering the main studies and clinical trials published to date [5,11,12,13,14,15,16,17,18,19,20], our results are consistent with patients with ABMR, where 60% of these patients presented positive or increased values coinciding with the diagnosis by biopsy and with the detection of DSA.

However, a finding that differentiates our study from others is that it describes dd-cfDNA as a better biomarker to predict TCMR than ABMR. Indeed, our data showed, in three of the four patients diagnosed with TCMR, that values of dd cfDNA > 1% at the third post-transplant month were maintained throughout the follow-up period regardless of the date of diagnosis of cell rejection (+4 months, +12 months, and +8 months, respectively), anticipating diagnosis by 1 to 3 months (data shown in Table 3). These patients showed the highest levels of dd-cfDNA in our study. In addition, in these three patients, the dd-cfDNA determination performed at 15 days post transplant was higher than 0.5%, a value that was considered an elevation. The only patient in this group (TCMR) who had levels below 1% was diagnosed with TCMR-IA, and this result is consistent with other studies, where they obtain values similar and even more similar to those obtained for kidney recipients with borderline rejection and, in this sense, it appears to have no positive predictive value for a borderline rejection or TCMR-IA. However, the results of Stites et al. [22] show that not all TCMR 1A and BR rejections are equal and that clinically adverse outcomes are associated with elevated dd-cfDNA levels despite all patients being managed the same way. In this sense, Aubert et al. [21], with a recent important and robust study with external validation, demonstrated that elevated levels of dd-cfDNA were highly associated with the presence, activity, and severity of all types of kidney allograft rejection, including subclinical rejection in recipients with stable allograft function. Given the small number of patients with BR or TCMR 1A included in our study, we cannot strongly contradict the cited studies.

With regard to the low plasma levels of dd-cfDNA in patients with no rejection signs, the most important studies on the predictive value of dd-cfDNA in kidney graft rejection, like DART (Diagnosis Acute Rejection in Kidney Transplant) [15], report that the high NPV of dd-cfDNA is the main factor to consider as a biomarker with an essential role in avoiding unnecessary biopsies. However, our results show a low NPV in the technique because other pathologies or biological post-transplant complications could also affect dd-cfDNA levels, and this must be considered when interpreting the result. Specifically, we found seven patients with no signs of rejection with dd-cfDNA values > 1%, among which the following were observed: two with obesity, three with recurrent autoimmune pathologies, and two with infections. Although, as Huang et al. mention [23], regarding the discrepancies described between the results of the histological and molecular techniques [24,25], these can be considered “false-positive” dd-cfDNA cases. An alternative explanation could be that these cases represent “false-negative” histology where the underlying injury is missed due to interobserver variability or sampling error. In addition to these considerations, the previously referred to study by Aubert et al. [21] also demonstrated an association with subclinical rejection in recipients with stable allograft function, contrary to previous studies [23], which reported that these patients remain stable over follow-up. This fact should be considered in our study, taking into account that, in our center, biopsies are not performed by protocol, and in fact, were only performed in one of seven of these patients, obtaining an inconclusive result for this sample.

On the other hand, some studies [5,11,12,13,14,15,16,19,20], in addition to relating dd-cfDNA levels with biopsy results and verifying that this biomarker turned out to be more effective than creatinine in defining clinical and subclinical rejection used in clinical routine, reported an association of dd-cfDNA values > 0.5%. In this sense, our results about the behavior of dd-cfDNA and creatinine (included in Table 3) are similar because they showed an increase in dd-cfDNA > 1% in five patients and dd-cfDNA > 0.5% in four patients either before or at the time of rejection diagnosis, while only two patients showed an increased in creatinine level in the same context. However, we only obtained a better PPV for TCRM.

In addition, concerning HLA antibodies, Obrisca and Botiu et al. [26,27] confirmed a strong association of dd-cfDNA with dnDSA and underlying alloimmune-mediated injury in renal allograft recipients in a cohort of patients with unsuspecting clinical characteristics for rejection and excellent allograft function. In this way, our combined analysis of dd-cfDNA and dnDSAs revealed that patients with ABMR (DSAs+) showed elevated dd-cfDNA levels > 0.5% in all post-transplant determinations, but we could not find a strong association between cfDNA > 1% values.

Nevertheless, dd-cfDNA could, together with other biomarkers in the context of a future score, make it possible to avoid invasive biopsies in most cases, especially when TCMR or ABMR are suspected. Also, as demonstrated by Halloran et al. [28], the combination of dd-cfDNA fraction and quantity was more potent than either dd-cfDNA fraction or quantity alone and validated a novel two-threshold algorithm incorporating both variables. In the same direction, Aubert et al. [21] demonstrated that the addition of dd-cfDNA, combined with the conventional markers used in kidney transplants to detect rejection, significantly increased the discriminative capability of rejection diagnosis. This opens up avenues to improve the management of renal transplant patients by more specifically tailoring the clinical indication of biopsies.

Regarding the strengths of our study, the strategy of prospective sample collection with storage until analysis enabled a retrospective review of clinical records to optimize resources (reagents) by prioritizing samples to be analyzed based on inclusion and exclusion criteria agreed upon by our multidisciplinary group.

However, we acknowledge that this study has some limitations. First, the small sample size is due to limited resources and the high cost of reagents. Furthermore, this study only considers patients transplanted in a single center and, therefore, only includes the ethnic subtypes present in the geographic area of southeastern Spain, and no external validation was performed. Second, the absence of protocol biopsies in our center resulted in 11 of 30 patients included in the study not having a biopsy performed and being included in the Banff 1 group as they did not present post-transplant complications related to renal function or rejection but with the uncertainty of underdiagnosing subclinical rejections. Third, in two patients with ABMR and another two patients with TCMR, the diagnosis was established mainly by clinical criteria since the biopsy samples were taken post treatment and, therefore, were not evaluable. It should be noted that neither of these two patients presented HLA antibodies. Fourth, some clinical data, such as those mentioned regarding the diagnosis of rejection, certain co-morbidities, or treatment adherence, are collected in the clinical history without a standardized protocol, so this has generated confusion among researchers and has required multidisciplinary meetings to unify these data in some patients.

## 5. Conclusions

We conclude that plasma levels of dd-cfDNA could be considered an additional or alternative biomarker for graft rejection monitoring in early post kidney transplant up to several months before its clinical presentation, especially for patients with suspected TCMR or ABMR. Our results show low values of sensitivity = 50%, specificity = 61.11%, rejection NPV = 64.71%, and rejection PPV = 46.13% of the technique, compared to reference values previously published. Only for TCRM did we observed better results for dd-cfDNA in these parameters than in creatinine, as well as the presence or absence of protocol biopsies. Our data suggest that dd-cfDNA could help to differentiate those patients with dnDSAs that are going to through rejection, better than creatinine, especially at 15 d post transplant. In the present study, it appears to have no positive predictive value for borderline rejection or TCMR IA. However, dd-cfDNA could, together with other biomarkers in the context of a future score, make it possible to avoid invasive biopsies in most cases, mainly in the case of suspected TCMR or ABMR.

## Figures and Tables

**Table 1:** The demographic and clinical characteristics of the transplant patients included in the study.

	NR (Banff 1)	BR (Banff 3)	ABMR (Banff 2)	TCMR (Banff 4)	Total
**Patients** (N, %)	18 (60%)	3 (10%)	5 (16.66%)	4 (13.33%)	30 (100%)
**Recipient Age** (**average mean** ± SD)	50.56 ± 11.75	46.67 ± 6.02	48.60 ± 5.85	46.50 ± 8.50	49.30 ± 9.93
**Recipient Sex** (% men/% women)	72.2/27.8	66.7/33.3	60/40	75/25	70/30
**Indication for Transplant** (N, %)					
DN	5 (16.7%)	0 (0%)	1 (3.3%)	2 (6.7%)	8 (26.7%)
IgAN	4 (13.3%)	0 (0%)	1 (3.3%)	1 (3.3%)	6 (20%)
LN	1 (3.3%)	0 (0%)	2 (6.7%)	0 (0%)	3 (10%)
GN	2 (6.7%)	0 (0%)	0 (0%)	0 (0%)	2 (6.7%)
PKD	2 (6.7%)	1(3.3%)	1(3.3%)	0 (0%)	4 (13.3%)
CKDu	4 (13.3%)	2 (6.7%)	0 (0%)	1 (3.3%)	7 (23.3%)
**Previous Infections** (N, %)					
Negative Serology	4 (13.3%)	0 (0%)	0 (0%)	0 (0%)	4 (13.3%)
CMV	7 (23.3%)	2(6.7%)	3 (10%)	1 (3.3%)	13 (%)
EBV	2 (6.7%)	0 (0%)	0 (0%)	0 (0%)	2 (6.7%)
BKV	1 (3.3%)	0 (0%)	1 (3.3%)	1 (3.3%)	3 (10%)
HIV	1 (3.3%)	0 (0%)	0 (0%)	0 (0%)	1 (3.3%)
CMV + EBV	3 (10%)	0 (0%)	0 (0%)	2 (6.7%)	5 (16.7%)
CMV + HIV	0 (0%)	0 (0%)	1 (3.3%)	0 (0%)	1 (3.3%)
CMV + BKV	0 (0%)	0 (0%)	0 (0%)	1 (3.3%)	1 (3.3%)
CMV + HSV	0 (0%)	1 (3.3%)	0 (0%)	0 (0%)	1 (3.3%)
EBV + HBV	1 (3.3%)	0 (0%)	0 (0%)	0 (0%)	1 (3.3%)
**Other Pathological History** (N, %)					
HBP	17 (56.6%)	2 (6.7%)	4 (13.3%)	4 (13.3%)	27 (90%)
Obesity	4 (13.3%)	0 (0%)	0 (0%)	2 (6.7%)	6 (20%)
DM	7 (23.3%)	1(3.3%)	2 (6.7%)	2 (6.7%)	12 (40%)
AI	4 (13.3%)	0 (0%)	2 (6.7%)	3 (10%)	9 (30%)
Hyperuricemia	5 (%)	1 (3.3%)	0 (0%)	1 (3.3%)	7 (23.3%)
**Preformed anti-HLA Antibodies** (N,%)	1 (3.3%)	1 (3.3%)	1 (3.3%)	0 (0%)	3 (10%)
**Previous Transplants** (N,%)	2 (6.7%)	1 (3.3%)	1 (3.3%)	0 (0%)	4 (13.3%)
**Previous Transfusions** (N,%)	1 (3.3%)	1 (3.3%)	1 (3.3%)	0 (0%)	3 (10%)
**Pregnancies** (N,%)	2 (6.7%)	1 (3.3%)	0 (0%)	0 (0%)	3 (10%)
**Type of Transplant** (N, %)					
Asystole	0 (0%)	1 (3.3%)	0(0%)	0 (0%)	1 (3.3%)
Brain Death	18 (60%)	2 (6.7%)	5 (16.6%)	4 (13.3%)	29 (96.6%)
**Donor–Recipient Incompatibilities (ABCDRDQ)** (**Average** ± SD)	6.8 ± 2.2	8.2 ± 1.4	6.6 ± 2.8	6.5 ± 0.6	7.0 ± 1.9
**Post-Transplant Therapy** (N, %)					
Tacrolimus	18 (60%)	3 (10%)	5 (16.7%)	4 (13.3%)	30 (100%)
Cyclosporine	13 (43.3%)	2 (6.7%)	5 (16.6%)	2 (6.7%)	22 (73.3%)
Everolimus	4 (13.3%)	1 (3.3%)	2 (6.7%)	1 (3.3%)	8 (26.6%)
MMF	3(10%)	0 (0%)	1 (3.3%)	0 (0%)	4 (13.3%)

NR, non-rejection; ABRM, antibody-mediated rejection; BR, borderline rejection; TCMR, T-cell-mediated rejection; SD, standard deviation; DN, diabetic nephropathy; IgAN, IgA nephropathy; LN, lupus nephropathy; GN, glomerulonephritis; PKD, polycystic kidney disease; CKDu, chronic kidney disease of unknown etiology; CMV, Cytomegalovirus; EBV, Epstein–Barr virus; BKV, BK virus; HIV, human immunodeficiency virus; HBV, hepatitis B virus; HSV, herpes simplex virus; DM, diabetes mellitus; HBP, high blood pressure; AI, autoimmune pathology; MMF, mycophenolate mofetil.

**Table 2:** Clinical post-transplant evolution of the patients included in the study.

	NR (Banff 1)	BR (Banff 3)	ABMR (Banff 2)	TCMR (Banff 4)	Total
**Patients** (N, %)	18 (60%)	3 (10%)	5 (16.66%)	4 (13.33%)	30 (100%)
**Post-transplant complications** (N, %)					
No adverse events	9 (30%)	0 (0%)	0 (0%)	0 (0%)	9 (30%)
Delayed graft function	4 (13.3%)	3 (10%)	0 (0%)	0 (0%)	7 (23.3%)
Acute humoral rejection	0 (0%)	0 (0%)	2 (6.7%)	0 (0%)	2 (6.7%)
Acute cellular rejection	0 (0%)	0 (0%)	2 (6.7%)	4 (13.3%)	6 (20%)
Borderline acute rejection	0 (0%)	3 (10%)	0 (0%)	0 (0%)	3 (10%)
Chronic humoral rejection	0 (0%)	0 (0%)	3 (10%)	0 (0%)	3 (10%)
Urinary tract infection	2 (6.7%)	0 (0%)	0 (0%)	0 (0%)	2 (6.7%)
Cyclosporine nephrotoxicity	0 (0%)	0 (0%)	0 (0%)	1 (3.3%)	1 (3.3%)
Tacrolimus neurotoxicity	0 (0%)	0 (0%)	1 (3.3%)	0 (0%)	1 (3.3%)
Interstitial nephritis	1 (3.3%)	0 (0%)	0 (0%)	0 (0%)	1 (3.3%)
Pyelonephrosis	1 (3.3%)	1 (3.3%)	0 (0%)	0 (0%)	2 (6.7%)
**Biopsies** (N, %)	7 (23.3%)	3 (10%)	7 (23.3%)	3 (10%)	20 (6.6%)
**dd-cfDNA (Average mean± SD, %)**	0.68 ± 0.79	0.35 ± 0.13	1.13 ± 1.31	2.12 ± 2.5	0.91 ± 1.32
**dd-cfDNA < 1%** (N, %)	11 (36.6%)	3 (10%)	2 (6.7%)	1 (3.3%)	17 (56.6%)
**dd-cfDNA ≥ 1%** (N, %)	7 (23.3%)	0 (0%)	3 (10%)	3 (10%)	13 (43.3%)
**Creatinine (Average±** SD, mg/mL)	1.63 ± 1.05	3.8 ± 1.7	2.16 ± 1.47	2.3 ± 1.24	2.03 ± 1.38
**Creatinine < 2** mg/mL	11 (36.6%)	0 (0%)	1 (3.3%)	2 (6.7%)	14 (46.6%)
**Creatinine ≥ 2** mg/ml	7 (23.3%)	3 (10%)	4 (13.3%)	2 (6.7%)	16 (53.3%)
**Post-transplant anti-HLA DSAs** (N, %)					
HLA-I	1 (3.3%)	0 (0%)	0 (0%)	0 (0%)	1 (3.3%)
HLA-II	4(13.3%)	0 (0%)	4 (13.3%)	0 (0%)	8 (26.7%)
HLA-I + HLA-II	0 (0%)	1 (3.3%)	0 (0%)	0 (0%)	1 (3.3%)
Non-HLA antibodies	0 (0%)	0 (0%)	1 (3.3%)	0 (0%)	1 (3.3%)

**Table 3:** Levels of dd-cfDNA and creatinine as rejection biomarkers, taking into account the time prior to the diagnosis of rejection for Group 3: ABMR (Banff 2) and TCMR (Banff 4).

Banff	ID	cfDNA +15 d	cfDNA +3 m	cfDNA +6 m	cfDNA +9 m	Cr + 15 d	Cr + 3 m	Cr + 6 m	Cr + 9 m	Rejection Date
2	TX011	0.7	0.44	0.79	1	2.67	0.99	1.04	1.18	9 m
2	TX012	5.6	2.7	2.4	2.2	3.58	1.41	1.25	1.55	1 m
2	TX027	0.77	0.08	0.06	0.08	6.53	3.06	4.66	1.87	10 d
2	TX096	2	0.55	0.64	0.45	1.34	1.41	1.62	1.46	2 y
2	TX110	0.33	0.48	0.81	0.59	3.83	1.19	1.79	0.94	1 y 9 m
4	TX004	0.54	1.3	1.5	0.83	1.74	1.68	3.58	3.22	4 m
4	TX014	0.28	0.35	0.46	0.66	2.29	3.12	4.08	5.42	6 m
4	TX022	0.7	1.8	5.40	3.50	2.09	1.32	1.39	1.46	1 y 9 m
4	TX023	0.5	1.5	9.30	5.30	1.36	1.23	1.05	1.79	8 m

**Table 4:** Results of validity study for dd-cfDNA as rejection biomarker, in general, and for each type of rejection.

	Rejection	BR	ABMR	TCRM
**Sensitivity (%)**	50	0	60	75
**Specificity (%)**	61.11	61.11	61.11	61.11
**PPV (%)**	46.15	0	30	30
**NPV (%)**	64.71	78.57	84.62	91.67

PPV: positive predictive value, NPV: negative predictive value, BR: borderline rejection, ABMR: antibody-mediated rejection, TCMR: T-cell-mediated rejection.

**Table 5:** Results of validity study for creatinine as rejection biomarkers for overall rejection and for each type of rejection.

	Rejection	BR	ABMR	TCRM
**Sensitivity (%)**	75	100	80	50
**Specificity (%)**	61.11	61.11	61.11	61.11
**PPV (%)**	56.25	30	36.36	22.22
**NPV (%)**	78.57	100	91.67	8462

PPV: positive predictive value, NPV: negative predictive value, BR: borderline rejection, ABMR: antibody-mediated rejection, TCMR: T-cell-mediated rejection.

## Data Availability

The data presented in this study are available on request from the corresponding author.

## Supplementary Materials

The following supporting information can be downloaded at: https://www.mdpi.com/article/10.3390/life14111491/s1, Table S1: the quantifications of isolated DNA.
