# Supplementary material for: Early Monitoring of Donor-Derived Cell-Free DNA in Kidney Allograft Recipients Followed-Up for Two Years: Experience of One Center
Source: Life (Basel). 2024 Nov 16;14(11):1491. doi: 10.3390/life14111491 (PMC11595986; doi:10.3390/life14111491)
Supplement: Supplementary file 1 [file life-14-01491-s001.zip › life-3289475-supplementary.pdf]

Supplementary Table S1.

		cf DNA concentration (ng/ul)			
		cfDNA (15 d.p.t)	cfDNA (3 m.p.t)	cfDNA (6 m.p.t	cfDNA (9 m.p.t
**Patient 1**	**TX001**	**3,07**	**0,29**	**8,07**	**1,32**
**Patient 2**	**TX003**	**2,56**	**1,12**	**1,21**	**1,59**
**Patient 3**	**TX004**	**2,7**	**0,83**	**1,62**	**1,89**
**Patient 4**	**TX006**	**1,3**	**1,8**	**2,05**	**1,8**
**Patient 5**	**TX007**	**1,56**	**24,1**	**1,52**	**1,96**
**Patient 6**	**TX008**	**0,83**	**1,27**	**0,42**	**120**
**Patient 7**	**TX009**	**1,3**	**1,48**	**1,6**	**1,04**
**Patient 8**	**TX010**	**1,94**	**1,95**	**58**	**1,52**
**Patient 9**	**TX011**	**9,66**	**3,28**	**3,15**	**4,44**
**Patient 10**	**TX012**	**2,19**	**1,24**	**0,67**	**0,78**
**Patient 11**	**TX014**	**3,27**	**0,78**	**0,86**	**2,21**
**Patient 12**	**TX015**	**1,88**	**1,56**	**2,03**	**1,95**
**Patient 13**	**TX016**	**2,6**	**1,8**	**3,8**	**2,8**
**Patient 14**	**TX018**	**2,5**	**2,25**	**32,3**	**6,88**
**Patient 15**	**TX022**	**1,11**	**1,6**	**1,8**	**2,1**
**Patient 16**	**TX023**	**0,6**	**1,2**	**1,8**	**2,1**
**Patient 17**	**TX026**	**0,7**	**1,2**	**1,14**	**2,34**
**Patient 18**	**TX027**	**3,64**	**6,07**	**5,8**	**17,7**
**Patient 19**	**TX028**	**1,28**	**4,75**	**0,88**	**2,38**
**Patient 20**	**TX029**	**1,35**	**1,76**	**1,76**	**1,09**
**Patient 21**	**TX030**	**2,83**	**3,1**	**4,15**	**3,47**
**Patient 22**	**TX031**	**0,83**	**1,23**	**2,01**	**2,6**
**Patient 23**	**TX032**	**2,2**	**0,57**	**1,91**	**1,01**
**Patient 24**	**TX034**	**0,92**	**1,76**	**1,85**	**2,59**
**Patient 25**	**TX035**	**2,83**	**1,95**	**1,72**	**3,08**
**Patient 26**	**TX047**	**9,67**	**2,7**	**1,3**	**0,88**
**Patient 27**	**TX070**	**2,89**	**1,46**	**2,66**	**1,19**
**Patient 28**	**TX094**	**4,42**	**1,68**	**1,26**	**1,56**
**Patient 29**	**TX096**	**1,5**	**0,71**	**2,68**	**1,36**
**Patient 30**	**TX110**	**2,19**	**13,2**	**4,17**	**2,09**
**mean**		2,54	2,96	5,14	6,59
**ds**		2,15	4,66	11,50	21,64
**median**		2,19	1,64	1,83	2,03

**p25**		1,30	1,21	1,36	1,40
**p75**		2,83	1,23	1,36	1,53
